# Prognostic impact of coronary microvascular dysfunction assessed by AMR in acute coronary syndrome patients with chronic kidney disease

**DOI:** 10.3389/fcvm.2024.1489403

**Published:** 2025-01-07

**Authors:** Ziyu Guo, Yike Li, Qiang Chen, Jingang Zheng

**Affiliations:** ^1^ Department of Cardiology, Peking University China-Japan Friendship School of Clinical Medicine, Beijing, China; ^2^ Department of Cardiology, China-Japan Friendship Hospital, Chinese Academy of Medical Sciences and Peking Union Medical College, Beijing, China; ^3^ Graduate School of Peking Union Medical College, Chinese Academy of Medical Sciences and Peking Union Medical College, Beijing, China; ^4^ Department of Cardiology, China-Japan Friendship Hospital, Beijing, China

**Keywords:** coronary microvascular dysfunction, angiography-derived microvascular resistance (AMR), acute coronary syndrome, chronic kidney disease, MACEs, all-cause mortality

## Abstract

**Background:**

Angiography-derived microcirculatory resistance (AMR) is proposed as a novel, pressure- temperature-wire-free and less-invasive method to evaluate coronary microvascular dysfunction (CMD). This study aims to examine the prognostic role of CMD assessed by AMR in predicting adverse events in acute coronary syndrome (ACS) patients with chronic kidney disease (CKD).

**Methods:**

This retrospective cohort study included ACS with CKD patients in the China-Japan Friendship Hospital from January 2016 to November 2022. The patients were divided into CMD and non-CMD groups based on AMR values of less than or greater than 250 mmHg*s/m.

**Results:**

A total of 345 eligible patients were included in this study. During a median follow-up of 23.0 months, higher prevalence rate of MACEs (28.3% vs. 15.1%, *P* = 0.003) and death (20.2% vs. 4.1%, *P* = 0.001) were observed in the CMD group. In multivariate Cox regression analysis, patients in the group of CMD had a 1.843 times higher hazard ratio (HR) for developing MACEs (HR: 1.843, 95% CI: 1.071–3.174, *P* = 0.027) and 5.325 times higher HR for developing death (HR: 5.325, 95% CI: 1.979–14.327, *P* < 0.001) for every 10 mmHg*s/m increment in AMR. The incorporation of AMR improved the predictive accuracy of the GRACE score for MACEs and death.

**Conclusion:**

This study indicates that the AMR is significantly related to poor prognosis among patients with ACS and CKD. Furthermore, AMR could improve the predictive power of the GRACE risk score. These results indicated that AMR may serve as a valuable clinical tool for classification, risk stratification or therapy individualization in these patients.

## Introduction

Chronic kidney disease (CKD) is well-known to be a global public health problem and is considered as an important independent risk factor for cardiovascular disease (CVD) development (1, 2). Meanwhile, CVD is a principal cause of death in CKD patients (3). Previous research found that up to 30%–40% of patients presenting with an acute coronary syndrome (ACS) could combine with CKD (4). Despite rapid progress in the treatment, ACS patients with CKD are still facing a high risk of unfavorable clinical outcomes. Traditional cardiovascular risk factors, such as diabetes, hypertension, metabolic abnormalities and aging are prevalent in ACS and CKD (5). These factors collectively contribute to endothelial cell damage, which in turn leads to coronary microvascular dysfunction (CMD). Several studies have highlighted that CMD is closely related to the adverse cardiovascular events of ACS and CKD (6–9). Despite its significance, limited research has been conducted on assessing abnormal coronary microcirculatory function or evaluating the predictive value of CMD in this patient population.

There are various approaches to assessing microvascular function, encompassing both non-invasive and invasive methods (10). Non-invasive methods included coronary computed tomography angiography (CCTA), cardiac magnetic resonance (CMR), and positron emission tomography (PET). Among these methods, cardiac PET is currently regarded as the gold standard for non-invasive assessment of coronary microvascular function (11); CMR has advantages of high-resolution and localization, which provides high diagnostic accuracy (12, 13). However, these methods are often constrained by high costs, the impracticality of repeated measures, or concerns about radiation exposure. Invasive methods include coronary angiography, Doppler flow map, and the index of microcirculatory resistance (IMR) (10). Among them, the thermodilution-based IMR is viewed as the gold standard for the invasive methods for its specificity to the microvasculature, greater quantitative precision, and its advantage of being unaffected by hemodynamic epicardial structural changes (14, 15). Many studies have demonstrated that IMR has diagnostic and prognostic values in a variety of clinical diseases (16–18). For instance, the study conducted by Fearon et al. demonstrated an IMR greater than 40 measured in patients with ST-Segment elevation myocardial infarction (STEMI) predicted adverse events, including rehospitalization, heart failure, or death (19). However, the need for a pressure-temperature sensor guide wire, the use of adenosine to achieve maximal hyperemia and the higher costs of IMR restricted its clinical application (20).

The angiography-derived microvascular resistance (AMR), a recently developed parameter, offers a simpler and more rapid assessment without the need for pressure wires and adenosine (21). There have several investigations examining the role of AMR and suggesting AMR as a valuable and reliable tool for diagnostic purposes. At the same time, AMR also has high predictive potential in a variety of clinical situations, including CAD, STEMI and myocardial infarction (MI) with non-obstructive coronary arteries (MINOCA) (14, 22–24). However, studies on the prognostic value of AMR in ACS patients with CKD have not yet been conducted. In addition, few studies assessed AMR in combination with other predictors, such as the GRACE score, which has a high predictive value in ACS patients. This study aimed to explore the connection between AMR and clinical outcomes and the prognosis value of AMRa and the incremental prognostic value of adding AMR into the GRACE score.

## Materials and methods

### Study population

This retrospective study consecutively enrolled 443 patients with ACS and CKD who have successfully undegone PCI at the China-Japan Friendship Hospital from January 18, 2016 to November 17, 2022. Inclusion criteria were as follows: (1) age 18–80 years (2) clinical diagnosis of ACS and CKD (3) successful completion of PCI. According to the current guidelines, ACS encompassed both STEMI and non-ST-segment elevation ACS (NSTE-ACS), the latter includes unstable angina (UA) and non-STEMI (25). According to the KDIGO guideline, CKD was defined as estimated glomerular filtration rate (eGFR) < 60 ml/min/1.73 m^2^ or the presence of albuminuria for at least 3 months, including uremia (26). Uremia was defined as eGFR < 15 ml/min/1.73 m^2^, the initiation of renal replacement therapy. The exclusion criteria were: (1) history of coronary artery bypass operation (2) heart failure (3) hemodynamic instability (4) severe coagulopathy disorders (5) malignant tumor. Additionally, the AMR exclusion criteria included (1) poor angiographic image quality (2) low contrast (3) unsatisfactory angiography view, and (4) severe distortion of the target vascular. After exclusions, 345 individuals with ACS and CKD were included. This study was approved by the Ethics Review Committee of China-Japan Friendship Hospital (No. 2020-112-K71) in compliance with the Declaration of Helsinki. Informed consent was waived because of the retrospective design.

### Data collection and definitions

Data of demographic and clinical information, serum biochemical parameters, and past medical history were collected. Demographic and clinical information comprised age, sex, body mass index (BMI), blood pressure (BP), heart rate and relevant comorbidities including hypertension, diabetes mellitus (DM), hyperlipidemia, etc. Laboratory measurements, such as white blood cell, platelet, hemoglobin, glucose, albumin and eGFR, and total cholesterol, were obtained. In addition, information on the use of medications, such as aspirin, P2Y12 inhibitors, β-blocker, and statins, was documented. Hypertension was defined as resting BP ≥ 140/90 mmHg or being on antihypertensive medications. DM was identified based on the use of blood glucose-lowering sugar or insulin, fasting plasma glucose levels ≥ 7.0 mmol/L or HbA1c ≥ 6.5% (27).

### Evaluation of quantitative flow ratio (QFR) and AMR

The analysis of QFR and AMR was conducted by using the AngioPlus system at the China-The calculation method and detailed interpretation of QFR have been described in previous’ s Japan Friendship Hospital by trained readers who were blinded to the outcome data. tudies (28, 29). In brief, the software automatically measured the blood vessel profile of the targeted coronary artery during contrast agent injection. The hyperemic flow velocity was calculated based on the centerline length divided by the time it takes to fill with the contrast agent. Next, an optimal vessel framework for analysis was selected depended on the adequate contrast agent filling and crisp lumen contour. The borders and main branches of the targeted vessel were automatically outlined. The reference vessel wall outline and diameter were then reconstructed according to the Murray bifurcation fractal law (30, 31). Finally, the pressure drop was calculated based on fluid dynamic equations and the distal coronary pressure (Pd) was calculated according to the pressure drop (29). QFR was calculated as Pd divided by the mean aortic pressure (Pa), while AMR was calculated as the ratio between Pd and the hyperemic flow velocity (Velocity_hyp_) (28). The vessel with the highest value of AMR among the patient's coronary arteries was selected. CMD was defined as an AMR ≥ 250 mmHg*s/m, following the definition established by Fan et al. (28).

### Follow-up and clinical outcomes

The median follow-up time was 23 months. The primary outcome was MACEs, as defined by a combination of nonfatal MI and all-cause mortality. Nonfatal MI was defined as an elevation of cardiac troponin values or creatine kinase-MB greater than the upper normal limit with at least one of the following: (1) the presence of typical MI symptoms, (2) pathological Q waves or ischemic changes on electrocardiogram, (3) severe coronary stenosis proved by angiography, and (4) regional wall motion abnormalities found on myocardium or echocardiography. All-cause mortality was defined as any death for any reason. The secondary outcome was all-cause mortality. Data collection on follow-up and outcomes was performed by experienced research nurses via telephone interviews, outpatient visits, or hospital records.

### Statistical analysis

For continuous variables, data were expressed as the mean ± standard deviation and tested by Student *t*-test unless otherwise stated. The categorical variables were expressed as count (%) and analyzed using Pearson's chi-squared (*χ*^2^) test or Fisher's exact test. The distribution of events over time was assessed by Kaplan-Meier (KM) survival curves and log-rank test. Cox proportional-hazards regression models were employed to investigate the relationship between AMR and clinical consequences in ACS with CKD patients. In Model 1, no adjustments were made to show the crude association. Model 2 was adjusted for age, dialysis, DM, systolic BP (SBP), heart rate (HR), eGFR, left ventricular ejection fractions (LVEF), Gensini score, Killip class ≥ II and invasive strategy. Model 3 was adjusted for Model 2 plus sex, BMI, hypertension, smoking, and prior MI. The relationships between AMR and study endpoints were further assessed using continuous scale with restricted cubic splines (RCS). To assess the predictive ability of AMR, the receiver operating characteristic (ROC) analysis was conducted. In addition, net reclassification improvement (NRI) and integrated discrimination improvement (IDI) were used to estimate the incremental predictive performance of outcomes after combining AMR with the GRACE score. A two-sided *P*-value < 0.05 was considered statistically significant. Statistical analysis was conducted with R software (version 4.2.0).

## Results

### Baseline characteristics

**Figure 1:**
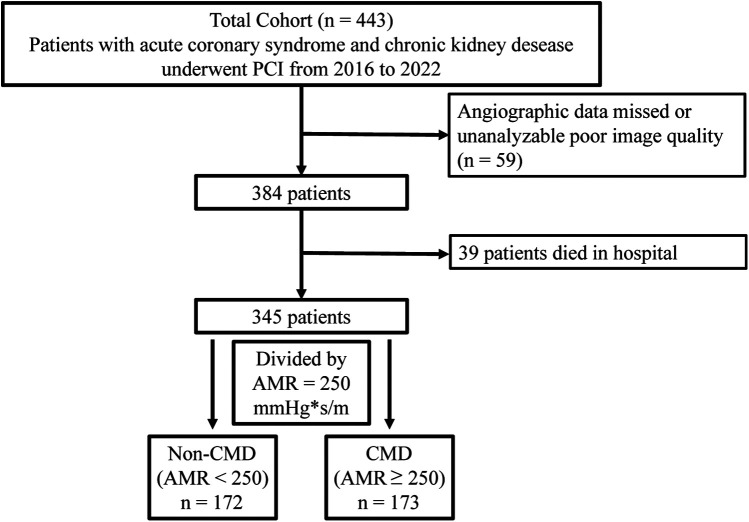
Flow chart of patient selection.

The flowchart of the participant enrollment is shown in Figure 1 and a total of 345 eligible patients were recruited. Detailed baseline clinical information, laboratory test, and medication use of this study are presented in Table 1. Overall, 31.3% patients were female and the average age was 68.14 ± 12.49 years; 144 (55.8%) reported smoking, 88.4% reported hypertension, 56.5% with diabetes, and 30.7% combined with hyperlipidemia. After assessing AMR, patients were divided into two groups: the CMD group (AMR ≥ 250 mmHg*s/m, *n* = 173) and the non-CMD (AMR < 250 mmHg*s/m, *n* = 172) group. The distribution of clinical features was similar between the two groups, such as the prevalence of hypertension, DM, MI, medication use, laboratory test, etc. However, QFR was significantly higher in the CMD group (0.94 ± 0.05 vs. 0.90 ± 0.05, *P* < 0.001), whereas the velocity of blood flow was significantly lower than in the non-CMD group (13.06 ± 2.36 cm/s vs. 18.33 ± 3.97 cm/s, *P* < 0.001).

**Table 1:** Baseline characteristics of the study participants.

Characteristic	Total (*n* = 345)	AMR < 250 (*n* = 172)	AMR ≥ 250 (*n* = 173)	*P-*value
General characteristics				
Age (years)	68.14 ± 12.49	67.58 ± 11.87	68.69 ± 13.08	0.411
Sex (female, *n*%)	108 (31.3)	52 (30.2)	56 (32.4)	0.755
BMI (kg/m^2^)	25.06 ± 3.91	24.94 ± 3.75	25.17 ± 4.06	0.579
Heart rate	78.12 ± 16.12	78.91 ± 14.78	77.33 ± 17.36	0.364
SBP (mmHg)	138.29 ± 21.98	139.44 ± 21.31	137.16 ± 22.64	0.336
DBP (mmHg)	77.83 ± 13.95	77.80 ± 12.73	77.86 ± 15.11	0.969
Comorbidities				
Hypertension (*n*%)	305 (88.4)	153 (89.0)	152 (87.9)	0.882
Diabetes (*n*%)	195 (56.5)	97 (56.4)	98 (56.6)	0.999
Hyperlipidemia	106 (30.7)	58 (33.7)	48 (27.7)	0.277
Smoking (*n*%)	144 (41.7)	78 (45.3)	66 (38.2)	0.213
COPD (*n*%)	9 (2.6)	3 (1.7)	6 (3.5)	0.505
KILLIP class ≥ II (*n*%)	143 (41.4)	77 (44.8)	66 (38.2)	0.255
Prior MI	81 (23.5)	35 (20.3)	46 (26.6)	0.215
Dialysis	37 (10.7)	21 (12.2)	16 (9.2)	0.475
Type of ACS				0.178
UA	147 (42.6)	80 (46.5)	67 (38.7)	
NSTEMI	150 (43.5)	72 (41.9)	78 (45.1)	
STEMI	48 (13.9)	20 (11.6)	28 (16.2)	
Invasive strategy	240 (69.6)	124 (72.1)	116 (67.1)	0.368
Laboratory values				
WBC (×10^9^/L)	7.72 ± 2.78	7.73 ± 2.68	7.71 ± 2.89	0.975
Lymphocyte (×10^9^/L)	1.38 ± 0.74	1.41 ± 0.78	1.36 ± 0.70	0.534
Platelets (×10^9^/L)	198.21 ± 63.32	201.02 ± 64.33	195.42 ± 62.37	0.413
Hemoglobin (g/L)	115.10 ± 20.96	115.17 ± 22.12	114.04 ± 19.75	0.347
Blood glucose (mmol/L)	8.45 ± 4.67	8.33 ± 4.45	8.56 ± 4.89	0.660
Albumin (g/L)	39.57 ± 4.90	39.49 ± 5.43	39.65 ± 4.33	0.756
eGFR (ml/min/1.73 m^2^)	28.77 ± 19.83	30.29 ± 19.91	27.25 ± 19.69	0.154
Total cholesterol (mmol/L)	3.97 ± 1.23	3.99 ± 1.27	3.96 ± 1.20	0.811
Triglyceride (mmol/L)	1.85 ± 1.12	1.90 ± 1.24	1.79 ± 1.00	0.349
HDL-c (mmol/L)	0.99 ± 0.28	0.97 ± 0.25	1.00 ± 0.31	0.291
LDL-c (mmol/L)	2.46 ± 0.96	2.46 ± 1.01	2.46 ± 0.91	0.950
LVEF (%)	55.16 ± 10.87	55.17 ± 10.88	55.15 ± 10.90	0.987
GRACE score	158.05 ± 34.51	156.20 ± 34.13	159.88 ± 34.89	0.322
Gensini score	55.00 ± 35.91	58.33 ± 34.28	51.69 ± 37.28	0.086
Medication (*n*%)				
ACEI/ARB	151 (43.8)	77 (44.8)	74 (42.8)	0.791
β-blockers	290 (84.1)	143 (83.1)	147 (85.0)	0.751
Statins	324 (94.2)	160 (93.0)	164 (95.3)	0.489
Aspirin	301 (87.2)	152 (88.4)	149 (86.1)	0.643
P2Y12 inhibitors	315 (91.3)	160 (93.0)	155 (89.6)	0.643
AMR (mmHg*s/m)	252.05 ± 48.19	216.40 ± 30.45	287.49 ± 34.47	<0.001
Flow velocity (cm/s)	15.69 ± 4.19	18.33 ± 3.97	13.06 ± 2.36	<0.001
QFR	0.92 ± 0.08	0.90 ± 0.09	0.94 ± 0.05	<0.001

Data are presented as number (%), mean ± SD, or median (interquartile range). BMI, body mass index; SBP, systolic blood pressure; DBP, diastolic blood pressure; COPD, chronic obstructive pulmonary disease; MI, myocardial infarction; ACS, acute coronary syndrome; UA, unstable angina; STEMI, ST-elevation myocardial infarction; WBC, white blood cell; eGFR, estimated glomerular filtration rate; HDL-C, high-density lipoprotein C; LDL-C, low-density lipoprotein C; LVEF, left ventricular ejection fraction; ACEI/ARB, angiotensin-converting-enzyme inhibitor/angiotensin receptor blocker; AMR, angiography-derived microcirculatory resistance; QFR, quantitative flow ratio.

### AMR and clinical outcomes

**Figure 2:**
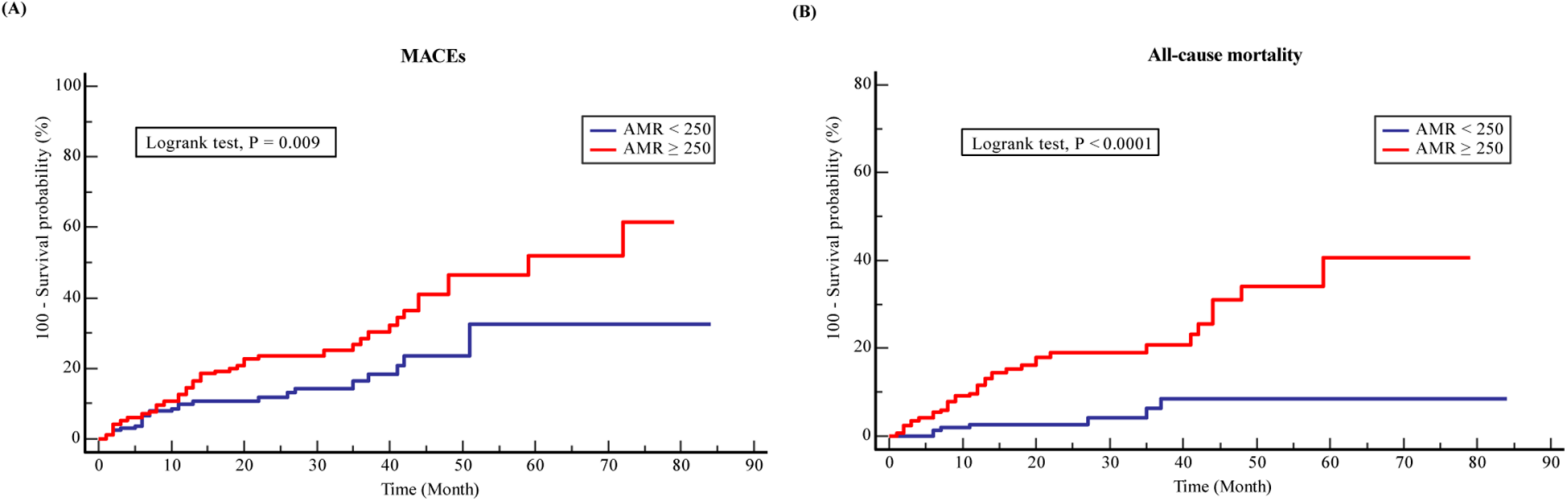
Kaplan-Meier analysis survival curves for **(A)** MACEs and **(B)** all-cause mortality in ACS with CKD patients.

Over the follow-up period, 75 (43.4%) MACEs and 42 (24.3%) all-cause mortality were recorded among the ACS and CKD patients. Higher incidence of MACEs (28.3% vs. 15.1%, *P* = 0.003) and all-cause death (20.2% vs. 4.1%, *P* = 0.001) was documented in the CMD group compared to the non-CMD group (Table 2). KM survival analysis stratified by AMR is presented in Figure 2. The KM survival curves demonstrate that the patients combined with CMD had a higher cumulative risk of the endpoints compared to those without CMD (log-rank test, all *P* < 0.05).

**Table 2:** Clinical outcomes according to AMR.

	AMR < 250 (*n* = 172)	AMR ≥ 250 (*n* = 173)	*χ* ^2^	*P*-value
MACEs, *n* (%)	26 (15.1)	49 (28.3)	8.843	0.003
All-cause death, *n* (%)	7 (4.1)	35 (20.2)	21.070	<0.001
Cardiac death, *n* (%)	3 (1.7)	24 (13.9)	17.588	<0.001
MI, *n* (%)	19 (11.0)	21 (12.1)	0.100	0.751
Unplanned revascularization, *n* (%)	48 (27.9)	23 (19.1)	3.745	0.053
Stroke, *n* (%)	7 (4.1)	7 (4.0)	0.000	0.991

MACEs, major adverse cardiovascular events; MI, myocardial infarction; AMR, angiography-derived microcirculatory resistance.

### Associations between AMR and mortality risk

Cox univariate and multivariate analysis was employed to evaluate the associations of AMR with adverse outcomes in ACS and CKD patients. Univariable Cox regression analysis indicated that AMR, SBP, HR, LVEF, KILLIP class ≥ II and prior MI, DM, and dialysis were statistically related to MACEs and every 10 mmHg*s/m increment in AMR linked to increased MACEs risk (HR: 1.095, 95% CI: 1.048–1.143, *P* < 0.001) (Supplementary Table S1). Utilizing the Multivariable Cox regression analysis by adjusting for variables with *P* < 0.05 (Model 2), AMR was proven to be a good predictor of the clinical endpoints in ACS with CKD patients and every 10 mmHg*s/m increase in AMR could bring an additional risk of incident MACEs (HR: 1.063, 95% CI: 1.018–1.111, *P* < 0.001) as well as all-cause mortality (HR: 1.123, 95% CI: 1.061–1.188, *P* < 0.001) (Table 3). After adjusting for additional confounders including sex, BMI, hypertension, smoking, and prior MI in Model 3, every 10 mmHg*s/m rise in AMR was connected to increased risk of MACEs events (HR: 1.065, 95% CI: 1.019–1.114, *P* = 0.005) and all-cause death events (HR: 1.139, 95% CI: 1.074–1.207, *P* < 0.001) (Table 3). It was interesting to note that this relationship was more pronounced in patients with AMR ≥ 250 mmHg*s/m than in normal-AMR patients. Every 10 mmHg*s/m increase in AMR showed a 1.843-fold adjusted increase risk for MACEs (HR: 1.843, 95% CI: 1.071–3.174, *P* = 0.027) and 5.325-fold adjusted increase risk for all-cause mortality (HR: 5.325, 95% CI: 1.979–14.327, *P* < 0.001) in patients with CMD (Table 3).

**Table 3:** Cox proportional hazards models for MACEs and all-cause mortality of patients for every 10  mmHg*s/m increase in AMR.

	Model 1		Model 2		Model 3	
	HR (95% CI)	*P*-value	HR (95% CI)	*P*-value	HR (95% CI)	*P*-value
All-cause death						
AMR < 250	Ref		Ref		Ref	
AMR ≥ 250	1.861 (1.157–2.995)	0.011	1.828 (1.071–3.121)	0.027	1.843 (1.071–3.174)	0.027
Continuous AMR	1.095 (1.048–1.143)	<0.001	1.063 (1.018–1.111)	<0.001	1.065 (1.019–1.114)	0.005
All-cause death						
AMR < 250	Ref		Ref		Ref	
AMR ≥ 250	4.952 (2.199–11.148)	<0.001	5.109 (1.916–13.622)	0.001	5.325 (1.979–14.327)	<0.001
Continuous AMR	1.173 (1.116–1.233)	<0.001	1.123 (1.061–1.188)	<0.001	1.139 (1.074–1.207)	<0.001

Model 1: unadjusted; Model 2: adjusted for age, dialysis, DM, SBP, HR, eGFR, LVEF, Gensini score, Killip class ≥ II, invasive strategy; Model 3: adjusted for age, sex, BMI, hypertension, DM, smoking, previous MI, dialysis, SBP, HR, eGFR, LVEF, Gensini score, Killip class ≥ II, invasive strategy.

Ref, reference; HR, hazard ratio; AMR, angiography-derived microcirculatory resistance.

**Figure 3:**
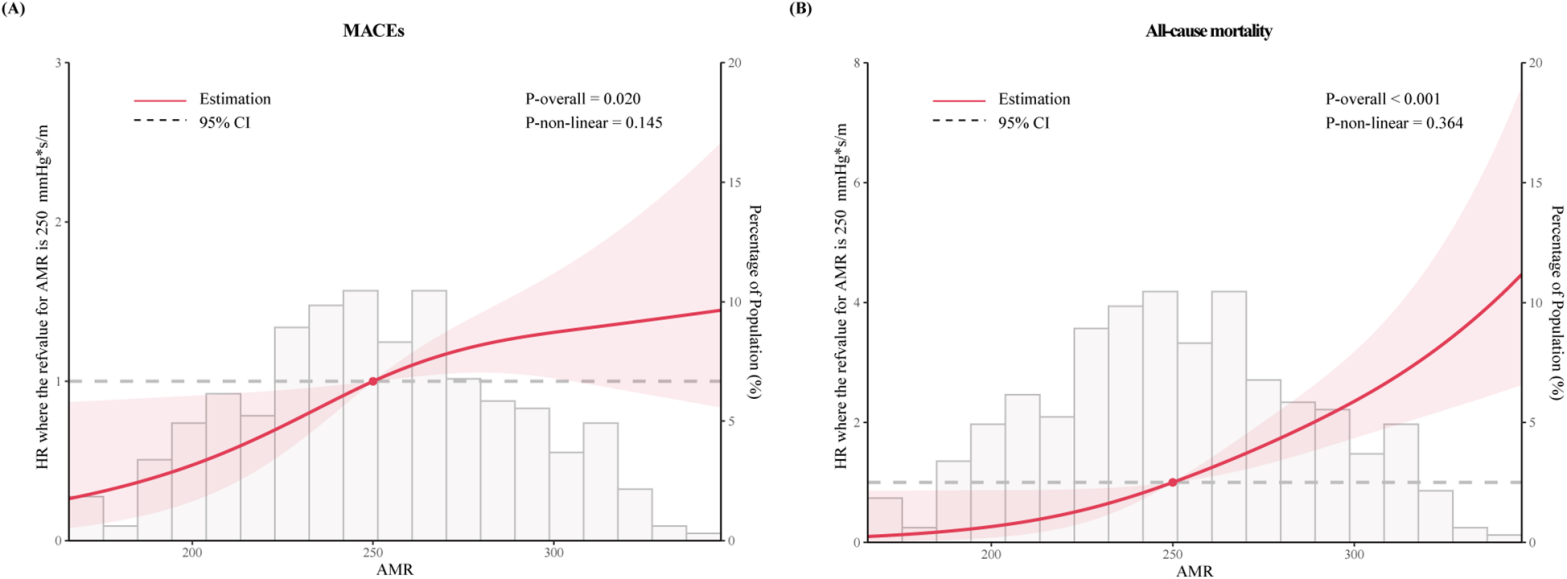
The restricted cubic spline for **(A)** MACEs and **(B)** all-cause mortality. The horizontal gray dashed line represents the HR = 1. Red lines represented references for hazard ratios, and red shaded areas represent 95% CI. Adjusted for for age, dialysis, DM, SBP, HR, eGFR, LVEF, Gensini score, Killip class ≥ II, invasive strategy. HR, hazard ratio.

RCS curves were utilized to visualize the trends and correlations between AMR and clinical outcomes (Figure 3). The spline curves revealed notable linear association between AMR and MACEs (*P* for nonlinearity = 0.145) as well as all-cause mortality (*P* for nonlinearity = 0.364), indicating that higher AMR was positively correlated with increased mortality, thereby suggesting a detrimental impact of elevated AMR on the lifespan of ACS patients with CKD.

### The incremental predictive value of AMR

**Figure 4:**
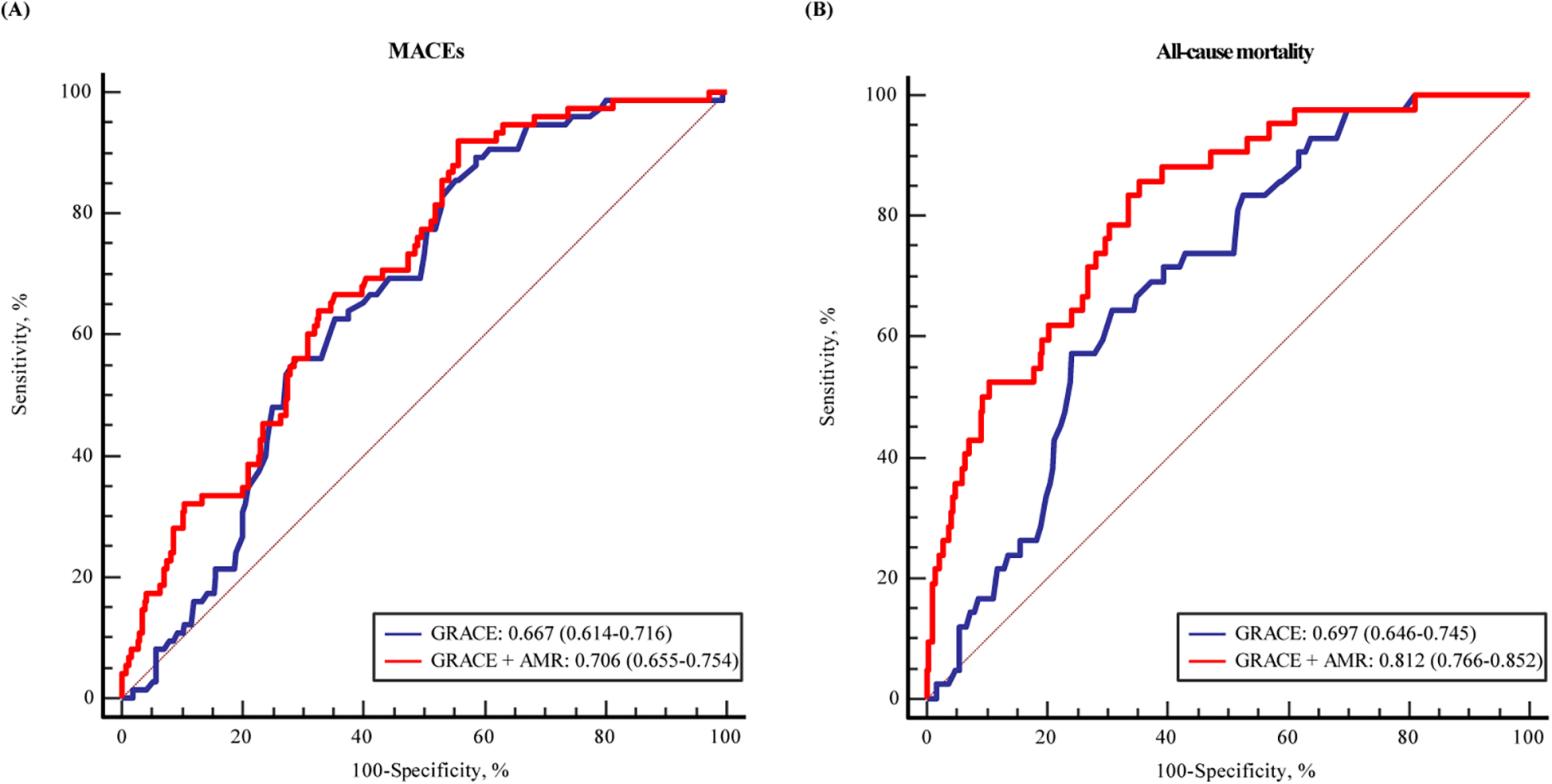
The ROC curve for the predicting **(A)** MACEs and **(B)** all-cause mortality by GRACE score and with addition of AMR in multiple logistic regression modelling.

The ROC curves and area under the curve (AUC) revealed that AMR could provide significant predictive value for MACEs (AUC: 0.636, 95% CI: 0.566–0.707, *P* < 0.001) and all-cause mortality (AUC: 0.763, 95% CI: 0.687–0.839, *P* < 0.001) in patients (Supplementary Figure S1). Further analysis were conducted to assess whether AMR had incremental predictive capacity for MACEs in ACS with CKD patients (Table 4). The results revealed that adding AMR to the GRACE score enhanced the capacity to predict MACEs, as shown by an increased AUC from 0.667 to 0.706 (*P* < 0.001) (Figure 4), an improvement in the C-statistic from 0.667 to 0.706 (Table 4). We evaluated improvements in risk stratification using the NRI and the IDI and found the incorporation of AMR into the GRACE score model resulted in an increase in the NRI (0.162, 95% CI: 0.008–0.339, *P* = 0.02) and IDI (0.040, 95% CI: 0.006–0.008, *P* < 0.01). Furthermore, adding AMR to the GRACE score improved the capacity and accuracy of predicting all-cause mortality, with an increased AUC from 0.697 (95% CI: 0.646–0.745) to 0.812 (95 % CI: 0.766–0.852) (*P* < 0.001) (Figure 4), significant improvement in the C-statistic (increasing from 0.697 to 0.812), NRI (0.288, 95% CI: 0.069–0.479, *P* = 0.01) and IDI (0.105, 95% CI: 0.040–0.176, *P* < 0.01) (Table 4).

**Table 4:** Model improvement for the AMR in combination with GRACE.

	C-index (95%CI)	*P*-value	Continuous NRI (95% CI)	*P*-value	IDI (95% CI)	*P*-value
MACEs						
Grace score	0.667 (0.614–0.716)	<0.01	ref	ref	ref	ref
Grace score + AMR	0.706 (0.655–0.754)	0.12	0.162 (0.008–0.339)	0.02	0.040 (0.006–0.088)	<0.01
All-cause death						
Grace score	0.697 (0.646–0.745)	<0.01	ref	ref	ref	ref
Grace score + AMR	0.812 (0.766–0.852)	<0.01	0.288 (0.069–0.479)	0.01	0.105 (0.040–0.176)	<0.01

MACEs, major adverse cardiovascular events; Ref, reference; AMR, angiography-derived microcirculatory resistance; GRACE, global registry of acute coronary events risk score; NRI, net reclassification index; IDI, Integrated discrimination improvement; CI, confidence interval.

## Discussion

This study first investigated the predictive value of AMR in ACS patients with CKD undergoing PCI. Our results suggested that AMR was independently related to MACEs and all-cause mortality in patients with ACS and CKD. The risk of MACEs and all-cause mortality significantly increased when AMR was ≥ 250 mmHg*s/m. Furthermore, the addition of AMR could improve the predictive value of the GRACE score to predict MACEs and all-cause mortality.

CMD is prevalent among ACS and CKD patients and it is proven to be a significant predictor for short- and long-term clinical outcomes (32–34). De Vita et al. found that ACS exhibited considerable coronary dysfunction, involving both an increased coronary microcirculation vasoconstrictor function and a decreased dilator function (35). CMD is also prevalent in CKD patients (36). Chronic kidney disease (CKD) creates a persistent systemic proinflammatory state that drives endothelial dysfunction, ultimately resulting in coronary microcirculatory impairment. This proinflammatory environment in CKD also induces vascular and myocardial adaptations and remodeling, which contribute to the development of vascular aging and calcification, and myocardial fibrosis (37). These consequences can appear as structural anomalies of the coronary microvasculature, which might result in CMD. Moreover, microvascular rarefaction in CKD patients, combined with endothelial dysfunction and reduced myocardial perfusion, in addition to the left ventricular hypertrophy and diastolic dysfunction, lead to compensatory vasodilation of arterioles and resting coronary flow elevation, and diminished coronary circulatory reserve eventually (5). A further study discovered CMD may mediate the impact of CKD on abnormal cardiac function and cardiovascular events in those without evident coronary artery disease (7). However, patients with CKD were excluded or accounted for only a relatively small part of studies. There is minimal evidence to explore the correlation between CMD and poor prognosis in ACS patients with CKD (38).

IMR, a traditional CMD measurement, is based on thermodilution-pressure wire. However, its clinical application is largely limited by the use of hyperemic agents, the requirement for a pressure wire, longer operation time and the higher cost (39). To overcome these barriers, a coronary angiography images-based calculation of microvascular resistance (AMR) has been proposed (39, 40). By the computational fluid dynamics (CPFD) method, AMR could be evaluated within 1 min, and the entire measurement process takes less than 5 min, allowing for the simultaneous identification of microcirculatory dysfunction during angiographic procedures (41, 42). Several research has proven a diagnostic value of AMR in 56 patients with no obstructive coronary arteries, with 84.2% accuracy, 86.1% sensitivity and 81.0% specificity, respectively, and confirmed that AMR could serve as an independent predictor of adverse cardiovascular events (20, 21, 43). Multiple studies have already proven that AMR was strongly related to adverse events and had an outstanding predictive capacity for adverse outcomes in different cardiovascular diseases (14, 23, 24, 44). However, the impact on prognostic of AMR has not been studied for patients with ACS and CKD.

To our knowledge, this is currently the first study to evaluate the relationship between AMR and clinical prognosis among ACS with CKD patients. We discovered patients with CMD had a higher rate of adverse events and exhibited a significantly increased rate of unfavorable prognostic outcomes after accounting for conventional risk factors. It is very noteworthy that the mortality rate increased significantly in ACS with CKD patients with AMR ≥ 250 mmHg*s/m in our study. Considering the high mortality rate in cases of ACS and CKD, our findings remind clinicians to pay more attention to the clinical management of these patients with CMD or AMR ≥ 250 mmHg*s/m. In addition, our findings revealed that adding the AMR to the GRACE score enhanced its predictive ability and accuracy for MACEs and all-cause mortality, as improvements in C-statistics, NRI and IDI. Our study focused on those with ACS and CKD, who are at a greater risk of overt cardiac dysfunction and a poor prognosis. The results of this study provide new views on the predictive value of AMR for patient outcomes. Taken together, AMR may give extra information about high-risk patients, assisting in the management or prevention of adverse outcomes. Meanwhile, AMR has the potential to increase the use of coronary microvascular function assessments while reducing the use of a pressure-temperature wire and technical errors.

In addition, prognostic stratification using AMR could be useful in many cardiovascular diseases, especially cardiovascular diseases with CMD. For example, MINOCA, a special type of MI, features clinical documentation of an acute MI without angiographically evident obstructive coronary artery obstruction (stenosis < 50%) (45). As an important mechanism, CMD plays an important role in patients with symptoms and/or signs of MI and non-obstructive coronary artery disease, including MINOCA (46). A retrospective multicenter cohort study concentrated on patients with MINOCA conducted by Ciliberti et al. revealed that nearly one out of two patients showed atherosclerosis progression, often requiring revascularization (47). Likewise, a large systemic review that included a total of 55,369 suspected MINOCA participants conducted by Simeone et al. has found that MINOCA was fraught with high rates of mortality, high readmission rates, and socioeconomic burden (48). Over the past few years, the incidence of MACEs in MINOCA patients has increased (49). Our study explores the clinical significance of AMR for assessing coronary microvascular function in a specific group of people. Given the importance of coronary microvascular function, AMR has important prognostic implications and potential therapeutic implications. Assessing the AMR in risk stratification in MINOCA patients is of guiding significance in clinical practice, considering the incidence of total adverse events increasing year by year, as well as the potential therapeutic and prognostic implication of the AMR. More research is needed to extend our findings to other patient populations.

This study still has certain limitations. First, this was a single-center retrospective cohort study, which might cause possible recall bias and be affected by lost follow-up. As a single-center study with a relatively small sample size, additional prospective, large-scale, multi-center investigations are needed to verify our findings. Second, not all coronary angiography images were appropriate for analysis, which may cause possible selection bias. Third, we did not quantify myocardial infarction area or other factors affecting the coronary microcirculation such myocardial bridging (50), which limit any further exploration of the association between AMR and prognosis. Fourth, the majority of the follow-ups were done over the phone or with medical records of readmission, which may be impacted by family economic status or the COVID-19 epidemic. Consequently, we lack data on coronary re-examinations one-year post-MI. Finally, this study was limited to the Chinese ACS with CKD patients, which may restrict the applicability of these findings to other races.

## Conclusion

Our findings indicated that AMR was independently associated with an increased risk of MACEs and all-cause mortality in patients with ACS and CKD. Furthermore, adding AMR to the GRACE score could improve the predictive value of MACEs and all-cause mortality. More studies are needed to confirm our findings and assess its predictive value in other cardiovascular diseases to increase its clinical usefulness.

## Data Availability

The raw data supporting the conclusions of this article will be made available by the authors, without undue reservation.

## Supplementary material

Supplementary Figure S1 The ROC curves for the of AMR for predicting **(A)** MACEs and **(B)** all-cause mortality during follow-up.
