# Supplementary figures and images for: Prognostic impact of coronary microvascular dysfunction assessed by AMR in acute coronary syndrome patients with chronic kidney disease
Source: Front Cardiovasc Med. 2025 Jan 7;11:1489403. doi: 10.3389/fcvm.2024.1489403 (PMC11746910; doi:10.3389/fcvm.2024.1489403)

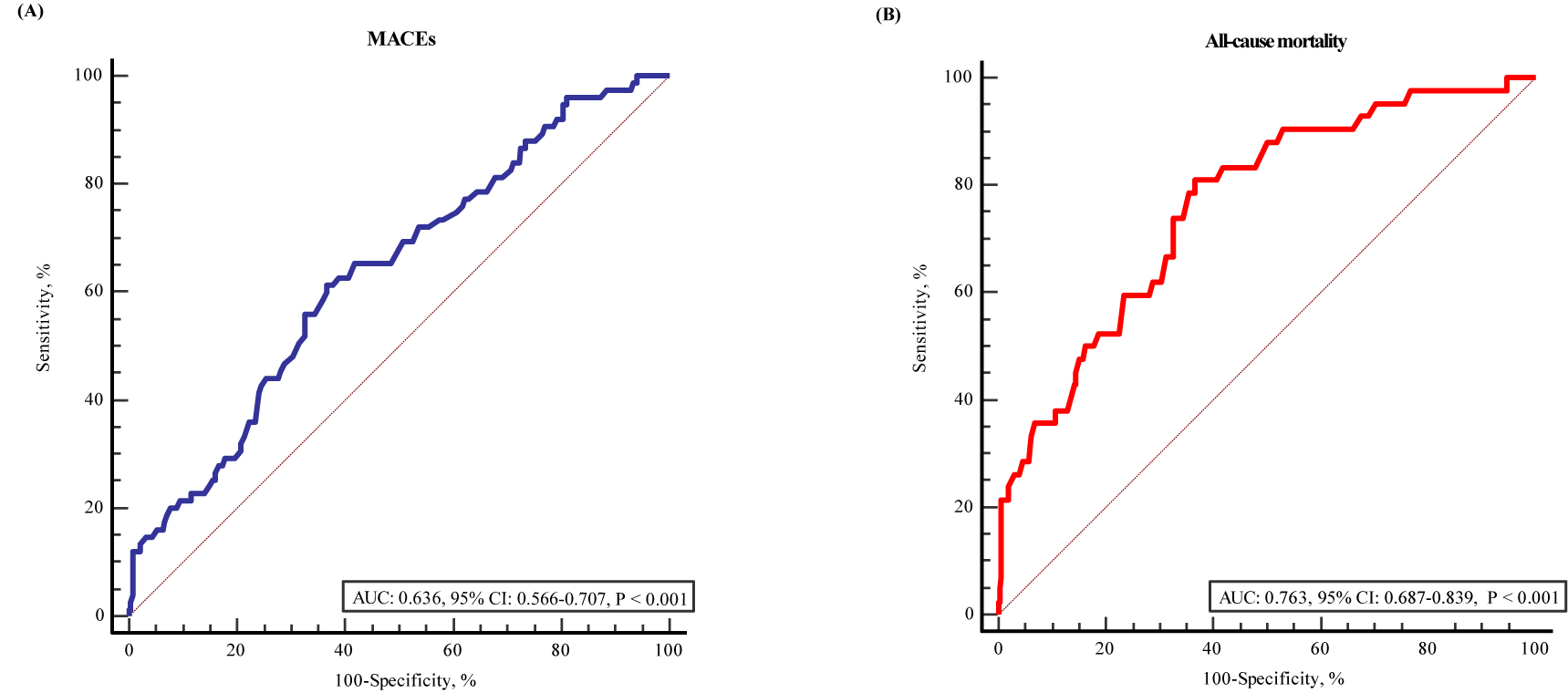

Supplement: Supplementary Figure S1 — The ROC curves for the of AMR for predicting (A) MACEs and (B) all-cause mortality during follow-up. [file Image1.tif]
