# Supplementary material for: Prognostic impact of coronary microvascular dysfunction assessed by AMR in acute coronary syndrome patients with chronic kidney disease
Source: Front Cardiovasc Med. 2025 Jan 7;11:1489403. doi: 10.3389/fcvm.2024.1489403 (PMC11746910; doi:10.3389/fcvm.2024.1489403)
Supplement: Supplementary file 2 [file Table1.pdf]

Supplementary Table 1 Association between abnormal AMR and clinical risk factors in patients with ACS and CKD

	Univariate analysis HR (95% CI)	*P*-value
Age	1.002 (0.983-1.022)	0.806
Female	0.882 (0.544-1.430)	0.612
BMI	0.968 (0.916-1.022)	0.243
Heart rate	1.025 (1.012-1.037)	< 0.001
SBP	1.012 (1.002-1.023)	0.021
Smoking	1.276 (0.801-2.030)	0.305
Prior MI	0.982 (0.578-1.669)	0.947
Hypertension	1.553 (0.625-3.859)	0.343
Diabetes	1.672 (1.029-2.717)	0.038
Dialysis	2.118 (1.073-4.182)	0.031
Gensini score	1.005 (0.999-1.011)	0.095
LVEF	0.975 (0.955-0.996)	0.019
KILLIP class ≥ II	2.259 (1.418- 3.600)	< 0.001
Invasive strategy	0.594 (0.371-0.951)	0.030
TC	0.871 (0.719-1.054)	0.156
TG	0.918 (0.742-1.135)	0.429
HDL-C	1.082 (0.490-2.391)	0.845
LDL-C	0.848 (0.663-1.084)	0.188
eGFR	0.980 (0.968-0.993)	0.002
AMR	1.095 (1.048-1.143)	< 0.001
AMR ≥ 250	1.861 (1.157-2.995)	0.011
ACEI/ARB	0.944 (0.597- 1.492)	0.803
β-blockers	1.923 (0.883-4.188)	0.100
Statins	1.547 (0.487-4.915)	0.460

BMI, body mass index; SBP, systolic blood pressure; MI, myocardial infarction; LVEF, left ventricular ejection fraction; TC, total cholesterol; TG, triglyceride; HDL-C, high-density lipoprotein C; LDL-C, low-density lipoprotein C; eGFR, estimated glomerular filtration rate; AMR, angiography-derived microcirculatory resistance; ACEI/ARB, angiotensin-converting-enzyme inhibitor/angiotensin receptor blocker.
